# Experience of childbirth in first-time mothers of advanced age – a Norwegian population-based study

**DOI:** 10.1186/1471-2393-13-53

**Published:** 2013-02-27

**Authors:** Vigdis Aasheim, Ulla Waldenström, Svein Rasmussen, Erica Schytt

**Affiliations:** 1 Department of Women’s and Children’s Health, Karolinska Institutet, Stockholm, Sweden; 2 Centre for Evidence-Based Practice, Bergen University College, Møllendalsveien 6, Bergen, 5009, Norway; 3 Centre for Clinical Research Dalarna, Falun, Sweden; 4 Institute of Clinical Medicine, Department of Obstetrics and Gynecology, Haukeland University Hospital, Bergen, Norway

**Keywords:** Experience of childbirth, Maternal age, Primiparous, Postponement of childbirth

## Abstract

**Background:**

Delaying the first childbirth to an advanced age has increased significantly during the last decades, but little is known about older first time mothers’ experience of childbirth. This study investigates the associations between advanced maternal age in primiparous women and the postnatal assessment of childbirth.

**Methods:**

The study was based on the National Norwegian Mother and Child Cohort Study (MoBa) conducted by the Norwegian Institute of Public Health. Data on 30 065 nulliparous women recruited in the second trimester 1999–2008 were used. Three questionnaires were completed: around gestational week 17 and 30, and at 6 months postpartum. Medical data were retrieved from the national Medical Birth Register. Advanced age was defined as ≥32 years and the reference group as 25–31 years. Descriptive and multiple logistic regression analyses were conducted.

**Results:**

Primiparous women aged 32 years and above expressed more worry about the upcoming birth than the younger women (adjusted OR 1.13; 95% CI 1.06-1.21), and 6 months after the birth they had a slightly higher risk of having experienced childbirth as ‘worse than expected’ (adjusted OR 1.09; 95% CI 1.02-1.16). The difference in birth experience was explained by mode of delivery. Comparisons within subgroups defined by the same mode of delivery showed that the risk of a more negative birth experience in the older women only applied to those with a spontaneous vaginal birth (adjusted OR 1.12; 95% CI 1.02-1.22). In women delivered by cesarean section, the older more often than younger women rated childbirth as ‘better than expected’ (elective cesarean delivery: adjusted OR 1.36; 95% CI 1.01-1.85, emergency cesarean delivery: adjusted OR 1.38; 95% CI 1.03-1.84).

**Conclusion:**

Postponing childbirth to ≥32 years of age only marginally affected the experience of childbirth. Older women seemed to manage better than younger with having an operative delivery.

## Background

Delaying the first childbirth to an advanced reproductive age has increased significantly among women during the last decades [1]. Whereas the higher rates of medical complications in first-time mothers of advanced age are well described [1-5], less is known about their experience of childbirth. The significance of women’s negative experience of childbirth is illustrated by its association with severe psychological problems such as postpartum depressive symptoms [6-8] and post-traumatic stress disorder [9], and also with mother-infant bonding [10]. A negative experience may even affect future reproduction with fewer subsequent births or a longer interval to next birth [11,12], which is particularly problematic at an age when fecundity is in decline [13].

Several factors that may influence the childbirth experience are more prevalent in women of advanced age, such as instrumental vaginal delivery [2,4], emergency cesarean delivery [14], premature birth [2], and social background factors such as being single and unemployed [15,16]. Women’s feelings for the upcoming birth may also affect the childbirth experience [7,15,17-19], and these may vary by age but in what direction remains unclear. On the one hand, women of advanced age are more likely to worry for the up-coming birth [20] and to prefer a cesarean delivery [21]. On the other hand, a nationwide Swedish study showed that primiparous women of advanced age (≥35 years) had more positive feelings about the upcoming birth compared to a reference group aged 26–29 [22], but postnatally, these women rated their overall experience as more negative than the younger women.

In this study we used data from the Norwegian Mother and Child Cohort to further investigate the association between advanced maternal age in primiparous women and the postnatal assessment of the birth experience.

## Methods

Data were drawn from the population-based Norwegian Mother and Child Cohort Study (MoBa), carried out by the Department of Public Health. The MoBa study investigates socio-demographic, physical, genetic, and mental health exposure variables and outcomes in mothers and their children. The method has been described in detail in previous publications [23,24]. Norwegian-speaking women were recruited during 1999–2008 from all Norwegian hospitals and maternity units with more than 100 births annually. A postal invitation, which included an informed consent form (for participation, follow ups and data linkage to the Norwegian Medical Birth Register) and the first of six questionnaires, was sent out after the women had registered for a routine ultrasound examination at approximately 17 weeks of gestation. The current study is based on version 6 of the quality- assured data files, released in 2011. Data from three of the questionnaires were used and these were completed around gestational weeks 17 and 30, and 6 months after the birth. A letter of reminder was sent out after 2–3 weeks in cases of unreturned questionnaires. The first questionnaire (Q1) obtained information about socio-demographic background (education, civil status, native language, income, unemployment and smoking) and reproductive background (previous pregnancies, in-vitro-fertilization (IVF). The second questionnaire (Q2) asked about worry about the upcoming birth and wish for a cesarean delivery, and the third (Q3) about the experience of childbirth. Data on maternal age, parity, mode of delivery and infant outcomes (prematurity, neonatal transfer) were retrieved from the Norwegian Medical Birth Register, which covers all births and includes information from the standardized medical records used by all antenatal clinics and delivery units in Norway [25].

For the present study, only nulliparous women who had completed all the three questionnaires, including the question about childbirth experience, and who had complete data from the Medical Birth Register on parity and age were included. Nulliparity was defined as women who had not given birth; either to a live or stillborn infant after 21 weeks of pregnancy [26]. Representativeness of the sample was assessed by comparison of a sub-sample giving birth in 2003 with all primiparous women in the entire Norwegian birth cohort (data from the Medical Birth Register) from the same year, which was approximately half-way through data collection. The age distribution of maternal age in Norway has since then been the same.

Age was defined as maternal age at the time of giving birth. The definition of ‘advanced’ maternal age was also based on data from the entire Norwegian birth cohort from 2003. We chose to define the upper quartile (breakpoint 31/32 years) as advanced maternal age, i.e. women of 32 years and beyond, and the comparison group as all women between the lower (24/25 years) and upper quartile, i.e. women aged 25–31 years. There is no consensus regarding how to define ‘advanced’ maternal age [27] and studies therefore use different age cut-off [2,28,29]. As fecundity starts to decline and medical complications increase much earlier than the commonly used 35 years limit [5,30], the 31/32 years limit was considered relevant. We did not include women younger than 25 years in the reference group which would have biased the results since these women constitute a selected group with higher risk of negative exposure [16] and outcome, for instance psychological distress [31].

Negative feelings about the upcoming labour and birth were measured by the statements ‘I really worry about giving birth’, and ‘If I could choose I would have a cesarean delivery’. The response alternatives were dichotomized into agree (‘Agree completely’ + ‘Agree’), and disagree (‘Agree somewhat’+ ‘Disagree somewhat’ + ‘Disagree’ + ‘Disagree completely’).

The childbirth experience was measured at 6 months after the birth (Q3) using the question: ‘Did the birth go as you had expected?’ The response alternatives were trichotomized into; 1) better (‘No, it was better’), 2) worse (‘No, it was worse’), and 3) as expected/mixed feelings (‘Yes, as expected’ + ’Neither better nor worse’).

### Statistical analyses

Differences between 1) the age groups and 2) between a sub-sample giving birth in 2003 and all primiparous women aged >25 years in Norway in 2003 were assessed by Student’s t-test and chi-square tests, respectively. Associations between maternal age and feelings about the upcoming labour and memory of the childbirth experience, and between maternal age and potential confounders, were tested in bivariate analyses. Multiple logistic regression analyses were performed and statistically significant confounders were adjusted for. To avoid adjusting for the natural process of ageing, potential confounders were restricted to the following socioeconomic factors: smoking, single status, native language other than Norwegian and education. In a final analysis of risk factors for a ‘worse than expected’ experience of childbirth we constructed an interaction variable between maternal age and mode of delivery, and also divided the women of advanced age into two groups: 32–37 years and ≥38 years.

The results are presented as crude and adjusted odds ratios (OR) with 95% confidence intervals (CI) [32]. The analyses were conducted using SPSS 19 (SPSS, Inc., Chicago, IL). The study was approved by the appropriate Regional Committees for Ethics in Medical Research and the Norwegian data Inspectorate (S-97045).

## Results

**Figure 1:**
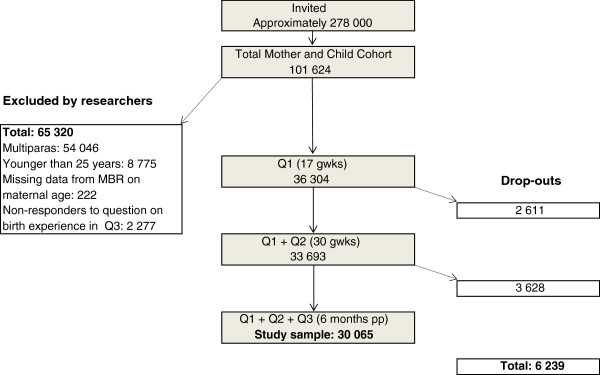
**Flow-chart of participants.** Recruitment, sample and dropouts. Q = Questionnaire. gwks = gestational weeks. pp = post partum. Drop-outs = non-responders to Q (2 611 + 2 858) or to question on birth experience (770).

The flow-chart (Figure 1) shows the total MoBa sample and our study group of 30 065 women, which remained after exclusion of multiparas, women younger than 25 years, missing data on age and childbirth experience, and drop-outs after Q1 and Q2. Table 1 shows that when compared with all Norwegian women who gave birth in 2003, the following characteristics were underrepresented in the sample: smoking, single status, IVF in present pregnancy, cesarean delivery, premature birth and infant transfer to neonatal intensive care unit. The table also shows that the following characteristics were more common in the older than in the younger women in our study: non-smoking, married or cohabiting, native language other than Norwegian, high income, a higher pre-gravid body mass index, IVF pregnancy, operative delivery, preterm birth and newborn transfer to neonatal unit.

**Table 1:** Background characteristics of primiparous women aged 25–31 years (reference group) and ≥32 years (advanced age), and for representativeness; comparisons between a sub-sample giving birth in 2003 and all primiparous women aged >25 years in Norway in 2003 (P-value for differences between a sub-sample from 2003 and all Norwegian primiparous women >25 years giving birth in 2003)

	**Women in the study**			**Women** ≥ **25 years giving birth in 2003**		
	**Age 25-31**	**Age **≥**32**	**All**	**Sub sample**	**Norway**	
	**n=21 226**	**n=8839**	**n=30 065**	**n=3549**	**n=16 760**	
	**%**	**%**	**%**	**%**	**%**	**P-value**
*Demographic and social factors*						
Age (years)*						0.07
25-29			50.8	55.2	54.0	
30-34			37.6	35.2	35.1	
35-39			10.3	8.8	9.6	
≥40			1.3	0.8	1.2	
Smoking*	8.4	7.6	8.2	10.8	13.9	<0.001
Single status	2.9	4.9	3.5	2.7	5.7	<0.001
Native language other than Norwegian	5.9	8	6.5	4.7		
Unemployed	1.7	1.6	1.7			
Income (NOR)**						
<200 000	11.2	6	8.7			
200 000–399 999	55.5	43.7	51.5			
≥400 000	29.1	46	33.7			
Pregravid BMI, mean (kg/m^2^)	23.6	24.1	23.8			
IVF present pregnancy*	2.6	8.9	4.4	3.8	4.9	<0.001
Labour*						
Mode of delivery						
Vacuum	14.2	16	14.8	14.1	13.7	0.54
Forceps	2.8	3.4	3	2.7	2.4	0.27
Elective cesarean delivery	3.1	6	4	3.8	5.3	<0.001
Emergency cesarian delivery	9.7	13.5	10.8	11.4	12.5	0.06
Unspecified cesarean delivery	1.9	2.9	2.2	1.2	2.5	<0.001
Prematurity**	5.5	6.4	5.8	6.5	8.8	<0.001
Neonatal Transfer	9.2	10.8	9.3	10.8	12.5	<0.001

*Data from the Norwegian Medical birth Register.

**NKR: NOK/GBP=10.90.

***Prematurity: 22–36 weeks of pregnancy.

**Figure 2:**
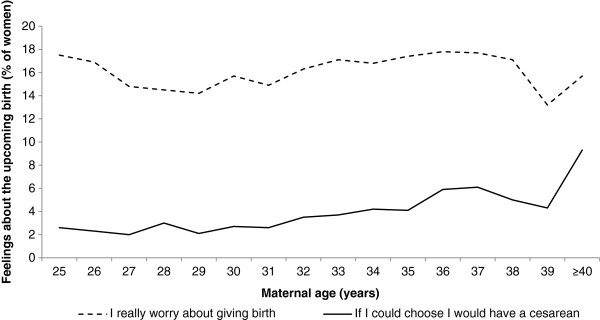
Nulliparous women's feelings about the upcoming birth in relation to maternal age when asked in gestational week 30 (n=30 065).

Figure 2 shows the percentages of women over the total age span from 25 to ≥40 years who agreed to the statements: ‘I really worry about giving birth’, and ‘If I could choose I would have a cesarean delivery’. The adjusted odds ratios for the respective outcome in women of ≥32 years compared with the younger reference group were 1.13 (95% CI 1.06-1.21) and 1.84 (95% CI 1.61-2.11) respectively.

**Figure 3:**
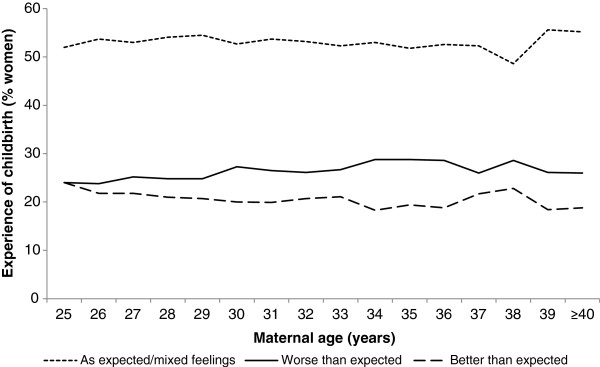
Experience of childbirth "as expected or mixed feelings", "better than expected" and "worse than expected" as remembered at 6 months postpartum by primiparous women of different age (n=30 065).

Figure 3 illustrates women’s childbirth experience at 6 months postpartum with the response ‘worse than expected’ increasing slightly by age and a corresponding decrease in the response ‘better than expected’. In the age group of ≥32 years, 27% reported the childbirth experience as ‘worse than expected’ compared with 25% in the reference group, a small but statistically significant difference (Table 2). When adjusting for mode of delivery the effect of age was no longer statistically significant (not shown). Table 2 also shows the four subgroups of women who had undergone the same mode of delivery, with comparisons between those of advanced age and the reference group. The prevalence of a ‘worse than expected’ experience was highest in women with emergency cesarean delivery regardless of age, followed by women with instrumental vaginal delivery, whereas the prevalence was lower after spontaneous vaginal birth and elective cesarean delivery. However, the *age-*related risk of a ‘worse than expected’ experience within each subgroup did not differ statistically, except in women with a spontaneous vaginal delivery where the oldest women were at higher risk (adjusted OR 1.12; 95% CI 1.02-1.22).

**Table 2:** Experience of childbirth in relation to expectations in primiparous women of advanced age (≥32 years) compared with the reference group (25–31 years)

**Maternal**	**Total**	**As expected/mixed feelings**				**Worse than expected**						**Better than expected**			
**Age, yrs**		**n**	**%**	**n**	**%**	**Crude OR**	**95% CI**	Adj OR*	**95% CI**	**n**	**%**	**Crude OR**	**95% CI**	Adj OR*	**95% CI**
						**All women**									
25-31	21,226	11,349	53.5	5376	25.3	1		1		4501	21.2	1		1	
≥32	8839	4652	52.6	2411	27.2	1.09	(1.03-1.16)	1.09	(1.02-1.16)	1776	20.1	0.96	(0.90-1.03)	0.97	(0.90-1.03)
					**Subgroups of women by mode of delivery**										
					*Women with a spontaneous vaginal birth*										
25-31	14,583	8268	56.7	2342	16.1	1		1		3973	27.2	1		1	
≥32	5192	2872	55.3	913	17.6	1.12	(1.03-1.23)	1.12	(1.02-1.22)	1407	27.1	1.02	(0.95-1.10)	1.02	(0.95-1.10)
					*Women with instrumentental vaginal delivery*										
25-31	3583	1668	46.6	1632	45.5	1		1		283	7.9	1		1	
≥32	1696	825	48.6	731	43.1	0.91	(0.80-1.02)	0.9	(0.80-1.02)	140	8.3	1.00	(0.80-1.25)	1.01	(0.81-1.26)
					*Women with instrumental vaginal delivery*										
25-31	2059	812	39.4	1,135	55.1	1		1		112	5.4	1		1	
≥32	1193	498	41.7	603	50.5	0.87	(0.75-1.01)	0.87	(0.74-1.01)	92	7.7	1.34	(1.00-1.80)	1.36	(1.01-1.85)
					*Women with elective cesarean delivery*										
25-31	665	477	71.7	66	9.9	1		1		122	18.8	1		1	
≥32	532	364	68.4	44	8.3	0.87	(0.58-1.31)	0.85	(0.56-1.30)	124	23.3	1.33	(1.00-1.77)	1.38	(1.03-1.84)

All women and subgroups of women by mode of delivery**.

Total sample = 30 065 women. Regression analyses presented as Crude and Adjusted Odds Ratio (OR) with 95% Confidence Intervals (CI).

*Adjusted for smoking, single status, native language other than Norwegian and education.

**Women with unclear mode of delivery or combined (VE and emergency cesarean delivery) excluded (n=562).

One in five women of advanced age (20%) had a ‘better than expected’ childbirth experience, which was similar to the comparison group (21%) (Table 2). The odds of having a ‘better than expected’ experience in the subgroups of women delivered by elective and emergency cesarean were increased in the older women compared with the younger.

To explore possible consequences of the way the question on childbirth experience was phrased, as being related to antenatal feelings, we also adjusted the analyses for *really worrying about the upcoming birth* and *a wish for a cesarean delivery*. The figures in Table 2 then remained almost unchanged (not shown).

As data from Table 2 suggested that older women tend to manage better than younger when exposed to an operative delivery we wanted to explore this issue further, and therefore divided the group of ‘advanced’ maternal age into two age groups, 32–37 years and ≥38 years (constituting the highest 2.5 percentile, labeled ‘very advanced maternal age’). Table 3, in which women aged 25–31 who had a spontaneous vaginal birth constitute the reference (=1) with which all other alternatives are compared, confirms that a ‘worse than expected’ experience decreased by maternal age and regardless of mode of delivery, even when adjusted for socio-demographic factors.

**Table 3:** **Memory of childbirth as "worse than expected" in primiparous women aged 32–37 and ≥38 years compared with the reference group aged 25**–**31 years, in relation to mode of delivery**

**Maternal age, yrs**	**n**	**%**	Adjusted OR*	**95% CI**
**Spontaneous vaginal birth**				
25-31 years	2342	16.1	1.00	
32-37 years	831	17.7	1.12	1.03-1.23
≥38 years	82	16.7	1.08	0.84-1.39
**Instrumental vaginal delivery**				
25-31 years	1602	45.3	3.41	3.13-3.70
32-37 years	639	43.3	3.14	2.79-3.52
≥38 years	83	43.2	3.03	2.25-4.08
**Emergency cesarean delivery**				
25-31 years	1135	55.1	4.89	4.42-5.41
32-37 years	519	51.4	4.29	3.74-4.92
≥38 years	84	45.7	3.75	2.75-5.13
**Elective cesarean delivery**				
25-31 years	66	9.9	0.48	0.37-0.63
32-37 years	37	8.6	0.44	0.31-0.62
≥38 years	7	7	0.34	0.15-0.74

Regression analyses presented as Adjusted Odds Ratio (OR) with 95% Confidence Intervals (CI).

*Adjusted for smoking, single status, native language other than Norwegian and Education.

## Discussion

This study showed that primiparous women of advanced age, defined as 32 years and beyond, were at a slightly higher risk of experiencing childbirth as ‘worse than expected’, compared with women aged 25–31 years. In pregnancy, they were also more often worried about the upcoming birth and if they could choose, they more often wished to have a cesarean delivery.

The association between advanced maternal age and a negative childbirth experience was no longer statistically significant after controlling for mode of delivery. We then assumed that the higher rate of emergency operative deliveries in older women, and the strong association between such deliveries and a negative experience of childbirth [15], explained this finding. However, the age effect in our study was the opposite: when exposed to an operative delivery, the older women seemed to manage better and reported more positive birth experiences than the younger. This trend was even more apparent when dividing the women into 32–37 years and ≥ 38 years. Reporting a birth experience as ‘worse than expected’ decreased with age in a dose–response manner, and women ≥38 years had the most positive experience. Older women may have been more aware of their age related risk of having an operative delivery and therefore been mentally more prepared [14,33], besides that the older women in beforehand were more positive to having a caesarean section. They may also have more mature strategies to manage a complicated birth because of earlier life experiences [33] and they may have felt relief after a prolonged [34,35] and complicated birth.

On the contrary, women of advanced age managed less well than the younger when having a spontaneous vaginal birth. Processes of biological ageing, such as decreased uterine function [36,37] as well as physical health [15], or prolonged labour [34], may have affected labour negatively, and consequently the childbirth was a difficult experience even if the birth technically was defined as normal, i.e. spontaneous vaginal birth. Additionally, induction of labour, which have previously been reported as a risk factor for a negative birth experience [15], was more prevalent in the women of advanced and very advanced maternal age who had an unassisted vaginal delivery than in the reference group (reference group 11.9%; advanced maternal age 14.3%; very advanced age; 23.4%) (p<0.001).

The higher rate of a ‘better than expected’ experience of childbirth in older women delivered by elective cesarean section has been reported previously [15]. A wish to have a cesarean delivery was more frequent in women of advanced age than in the reference group, and the fact that their preferences were met may be one explanation to the finding. Older women are also more often well prepared before childbirth [33], and it may be easier to prepare for a predictable event as an elective cesarean delivery than for labour and a vaginal birth.

These findings partly confirm the results from a previous national Swedish study of 1 302 first-time mothers. In agreement, the Swedish women of advanced age had a more negative birth experience than the younger, but contrary to the Norwegian women in the present study, more positive feelings prior to childbirth [22]. The survey questions were different in the two studies and also the time point for measurement differed; in the Swedish study expectations for the upcoming birth were measured in gestational week 17 and in the Norwegian, in gestational week 30. Optimistic expectations in early pregnancy may have altered towards more pessimistic or realistic after being informed through parental education, and finally facing the unknown. There may also be differences in the maternity care and childbirth preparations between the two countries. Further, from the present cohort, we have previously showed that women of advanced maternal age had an increased risk of psychological distress in terms of anxiety and depression during pregnancy and postpartum, which may have affected their assessments of childbirth experience [31]. In the smaller Swedish study, the older women were not at such a risk.

The strength of this study was the large sample size and age distribution similar to the national Norwegian birth cohort. Still the sample differed from the national cohort regarding characteristics that could have led to an underestimation of the risk for a negative childbirth experience, such as lower rates of single mothers, smokers, IVF pregnancies, preterm births and newborn transfers to neonatal intensive care unit. A limitation of this study was that data for the feelings for the upcoming birth and birth experience were restricted to responses to single items questions from the MoBa survey and not measured by validated instruments. The fact that the survey question included an assessment of childbirth experience relative to the woman’s expectations seemed to be of little importance since the results did not change after adjusting for worrying childbirth and for wishing a cesarean delivery.

## Conclusion

We conclude that delaying the first pregnancy to 32 years and older may have a negative effect on women’s childbirth experience, but only in cases of spontaneous vaginal birth. Even if women who are delivered by emergency cesarean section or instrumental vaginal delivery have a much more negative experience than after a spontaneous vaginal birth, older women seem to be better prepared to manage this experience than younger women. Whether primiparous women of advanced age would have a better experience after being informed about the possible risk of a prolonged labour needs further investigation.

### Ethics approval

The study was approved by the appropriate Regional Committees for Ethics in Medical Research and the Norwegian data Inspectorate (S-97045).

## Competing interests

The authors declare that they have no competing interests.

## Author’s contribution

VAA contributed to the planning of the study and analysed the data, contributed to the interpretation of findings and wrote the first draft of the manuscript. UW was the principal investigator and contributed with the idea, the interpretation of the results and with writing the manuscript. SR contributed in the analyses, and commented the manuscript. ES contributed to the planning of the study, to the data analyses, the interpretation of the results and the writing of the manuscript. All authors read and approved the final manuscript.

## Pre-publication history

The pre-publication history for this paper can be accessed here:

http://www.biomedcentral.com/1471-2393/13/53/prepub
